# From little things, big things grow: An exploratory analysis of the national cost of peripheral intravenous catheter insertion in Australian adult emergency care

**DOI:** 10.1111/1742-6723.14009

**Published:** 2022-05-14

**Authors:** Rachel Morgan, Emily Callander, Louise Cullen, Katie Walker, Suzanne Bumpstead, Tracey Hawkins, Lisa Kuhn, Diana Egerton‐Warburton

**Affiliations:** ^1^ School of Clinical Sciences at Monash Health Monash University Melbourne Victoria Australia; ^2^ Faculty of Medicine, Nursing and Health Sciences Monash University Melbourne Victoria Australia; ^3^ School of Public Health and Preventive Medicine Monash University Melbourne Victoria Australia; ^4^ Department of Emergency Medicine Royal Brisbane and Women's Hospital Brisbane Queensland Australia; ^5^ School of Public Health Queensland University of Technology Brisbane Queensland Australia; ^6^ School of Medicine The University of Queensland Brisbane Queensland Australia; ^7^ Department of Emergency Medicine Monash Health Melbourne Victoria Australia; ^8^ Monash Emergency Research Collaborative Monash Health Melbourne Victoria Australia; ^9^ School of Nursing and Midwifery Monash University Melbourne Victoria Australia

**Keywords:** Australia, emergency treatment, healthcare costs, medicine, peripheral venous catheterisation, staphylococcal infections

## Abstract

**Objective:**

To estimate the total economic impact of peripheral intravenous catheter (PIVC) or cannula insertion and use in adult Australian EDs, including those cannulas that remain unused for therapeutic purposes.

**Methods:**

Searches on Australian government websites were conducted to find rates of insertion, complications and cost of cannula; following this, gaps in national data sets were filled with MEDLINE and PubMed searches to estimate the total cost of cannula use in Australian EDs. Once the data were collected, totals were combined to establish an estimated cost for the listed categories.

**Results:**

The estimated cost of cannulation in Australia may be up to A$594 million per year, including the cost of insertion (equipment and staff), cost of complications such as *Staphylococcus aureus* bacteraemia and phlebitis, and patient‐centred costs (lost patient productivity, infiltration, occlusion and dislodgement). Approximately A$305.9 million is attributed to unused cannulas and approximately 11 790 days of clinician time is spent annually inserting cannula that remains idle.

**Conclusion:**

The figures developed in the present study represent an important educational opportunity to encourage thoughtful consideration of all interventions, no matter how small. ED cannula insertion represents a large economic and health cost to Australia's health system, many of which remain unused. There are no national data sets that record complications associated with PIVCs and we highlight the urgent need for improved data.

## Graphical abstract

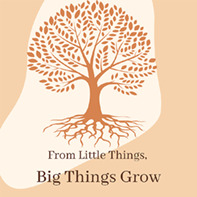

**Key findings**

- Cannula insertion represents a large economic and health cost to Australia's health system, costing over half a billion dollars a year.
- There is a lack of national data compiled on the complications of these prolific devices, which is needed to drive clinical change, prevent harm and minimise cost associated with cannula use.
- This study aims to provide an important educational opportunity to encourage thoughtful consideration of all interventions, no matter how small.

## Introduction

Peripheral intravenous catheter (PIVC) or cannula insertion is a common procedure in patients in ED settings; it has been estimated that up to half of patients have cannulation performed during their ED assessment and management.^1^ Insertion of cannulas is not risk‐free, with complications including pain, failed insertion, infiltration, occlusion and dislodgement which cause significant distress to the patient and possibly impact length of stay (LoS).^2^, ^3^, ^4^ One of the most serious complications is hospital‐acquired bacteraemia. This is associated with substantial economic costs to healthcare, in addition to the human costs of morbidity and mortality.^5^

The cost of managing cannulas is considerable when including the cost of equipment, time and complications associated with these devices.^5^, ^6^ Health systems rely on clinicians implementing necessary treatment and procedures for safe and effective patient management. Utilising cannulas appropriately is likely to be a small but effective way to decrease the economic and health burden on our healthcare system. Previously, the cost of cannulation has been determined using narrow parameters, for example, equipment cost in an Australian state, or the cost of admission and management of bacteraemia caused by cannula insertion.^5^, ^6^

The aim of this analysis was to estimate the overall annual cost of cannula insertion in adult care in Australian EDs. The costs incorporated into this analysis include equipment, staff time to insert, complications, lost patient productivity (days absent from work secondary to phlebitis or bacteraemia) and the opportunity cost of staff time taken to insert cannulas and the cost of unused devices.

## Methods

### Study design

The figures calculated in the present study used a combination of national data sets for raw data and previous research for any otherwise unattainable information, such as for cost and prevalence of insertion and associated complications to estimate the annual cost of cannulation in Australian EDs. Author EC provided valuable insight on the following methods as the health economist on the team. The literature search considered all papers up to May 2021.

To estimate the per annum economic cost of cannula insertions and complications, the combination of the number of annual Australian ED attendances by adults, all‐cannula insertion and all‐cannula complication rates derived from a literature review was used. The literature was examined to determine whether these complications increased bed stay (e.g. because of bacteraemia or phlebitis) or required replacement cannulation (e.g. because of infiltration, dislodgement or occlusion). For the complications that needed a cannula replaced, we added on the cost for another cannula insertion for the prevalence of that complication. For complications that increased LoS, the Independent Hospital Pricing Authority was used to estimate the additional cost per hospital bed day.^7^ Cannula insertion and complication rates were multiplied by the cost of cannulation to estimate the total amount spent on both equipment and staff per annum. This approach was then repeated for estimated unused cannula insertions and complication rates to assess the overhead cost of idle cannula in ED attendances.

### Ethics approval

This analysis was within the Quality Assurance and Evaluation guidelines and was exempt from the Human Research Ethics Committee ethical approval pathway.

### Outcomes

The primary outcome was cost including economic, and staff time. Secondary outcomes included the cost of cannula insertion alone, patient‐centred cost, complication cost (bacteraemia or phlebitis), rates of cannula insertion for 1 July 2019 to 30 June 2020, and time taken to insert a PIVC. Exploratory outcomes included the cost of unused cannulas in the categories listed.

### Data definitions

Data definitions used in the present study included:

- Phlebitis: one or more of pain, tenderness, redness or a palpable cord.^3^
- Adults: 19 years and older.^1^
- Unused cannulas: devices not used during ED admission for fluids or medication.^1^
- Patient‐centred costs: morbidity (days absent from work because of increased hospital LoS), and complications that do not affect LoS (infiltration, occlusion or dislodgement).

### Currency, price date and conversion

Currency, when not provided in Australian dollars (A$), was converted to this unit. All historical values have been updated using the Consumer Price Index calculator from the Reserve Bank of Australia for the 2020 calendar year.^8^

### Patient demographic characteristics and national data sets

Participants of interest included adults who presented to an Australian ED from 1 July 2019 to 30 June 2020. We searched on the Australian Institute of Health and Welfare (AIHW) and MyHospitals websites for Australia‐wide ED attendance statistics, number of cannulas inserted, use of these devices and the prevalence of cannula‐associated complications, as well as for the average cost of in‐hospital bed days.^7^, ^9^

The AIHW reports the number of public ED attendances per financial year, grouped by age with a group of 15–24 years old. To include all adults in our analysis, 15–18 years old from this age group were included.^9^ A total of 1428 *Staphylococcus aureus* bacteraemia (SAB) episodes hospital wide were recorded from 1 July 2019 to 30 June 2020.^10^ There were no data available regarding whether SAB episodes were attributable to PIVCs. Gaps in publicly published data were filled with the following literature search.

### Literature search

**Figure 1:**
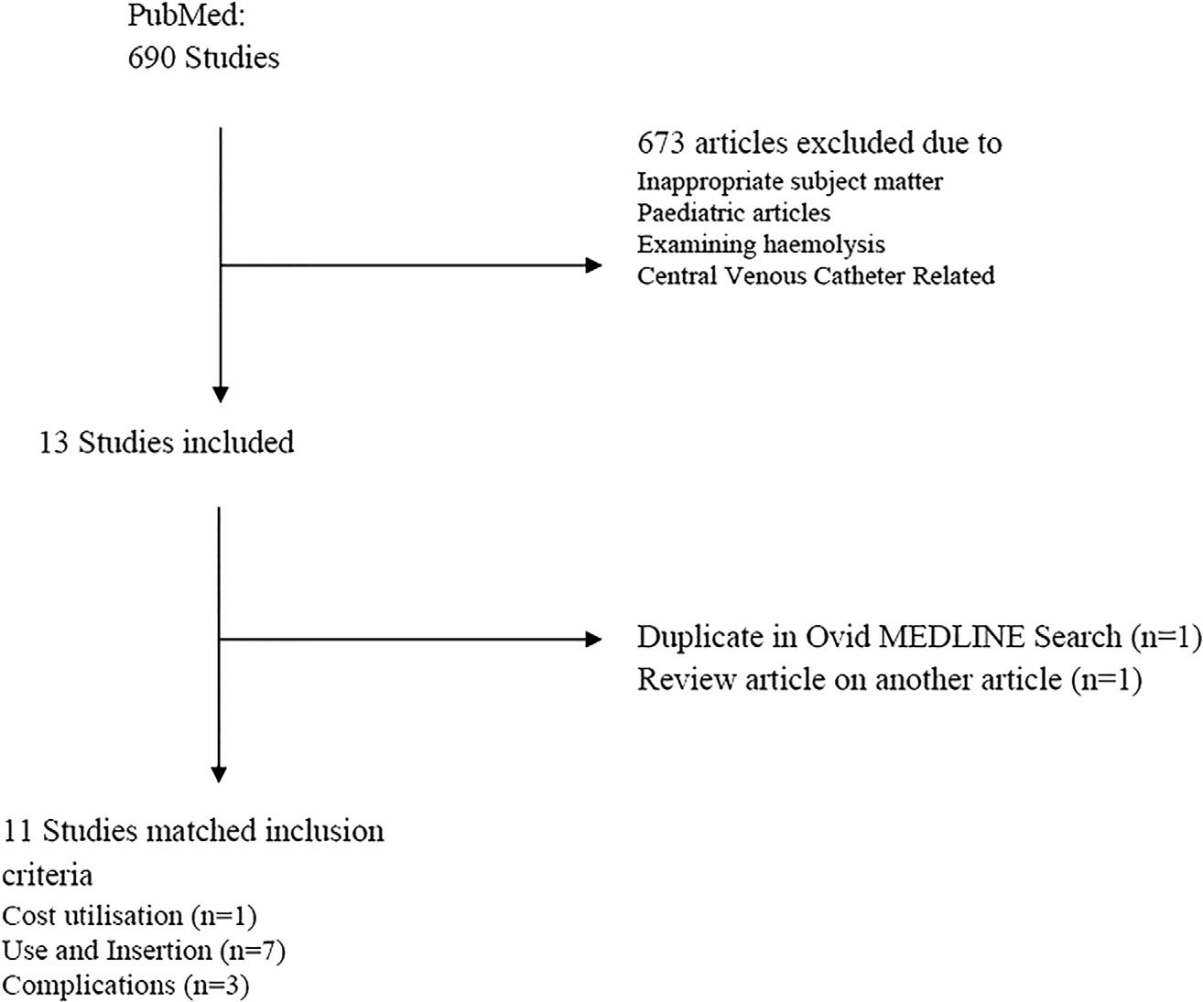
A PubMed literature search workflow describing exclusion and retention criteria for studies on cannula insertion rate data. Search terms included in Appendix S1.

Ovid MEDLINE and PubMed were used to find cannula insertion rates and cost (Appendix S1). Included studies reported cannula use in EDs in adults. Exclusion criteria were paediatric patients, and specifically the term ‘high flow’ to eliminate studies with ‘high flow nasal cannula’. A search for published papers on cannula use in Australia (Fig. 1) revealed that 36.0–42.1% of adults attending EDs had cannula inserted.^6^, ^10^, ^11^ In addition, 32.6–52.0% of ED cannula remain unused and were inserted ‘just in case’.^1^, ^6^, ^12^, ^13^ An Australian‐based research team found the cost of cannulation related to staff and equipment to be A$16.66 and A$6.69 per PIVC insertion, respectively.^6^, ^7^ Time cost was calculated by using pre‐existing research which reported that it took an average of 15.3 min to insert each cannula.^6^

**Figure 2:**
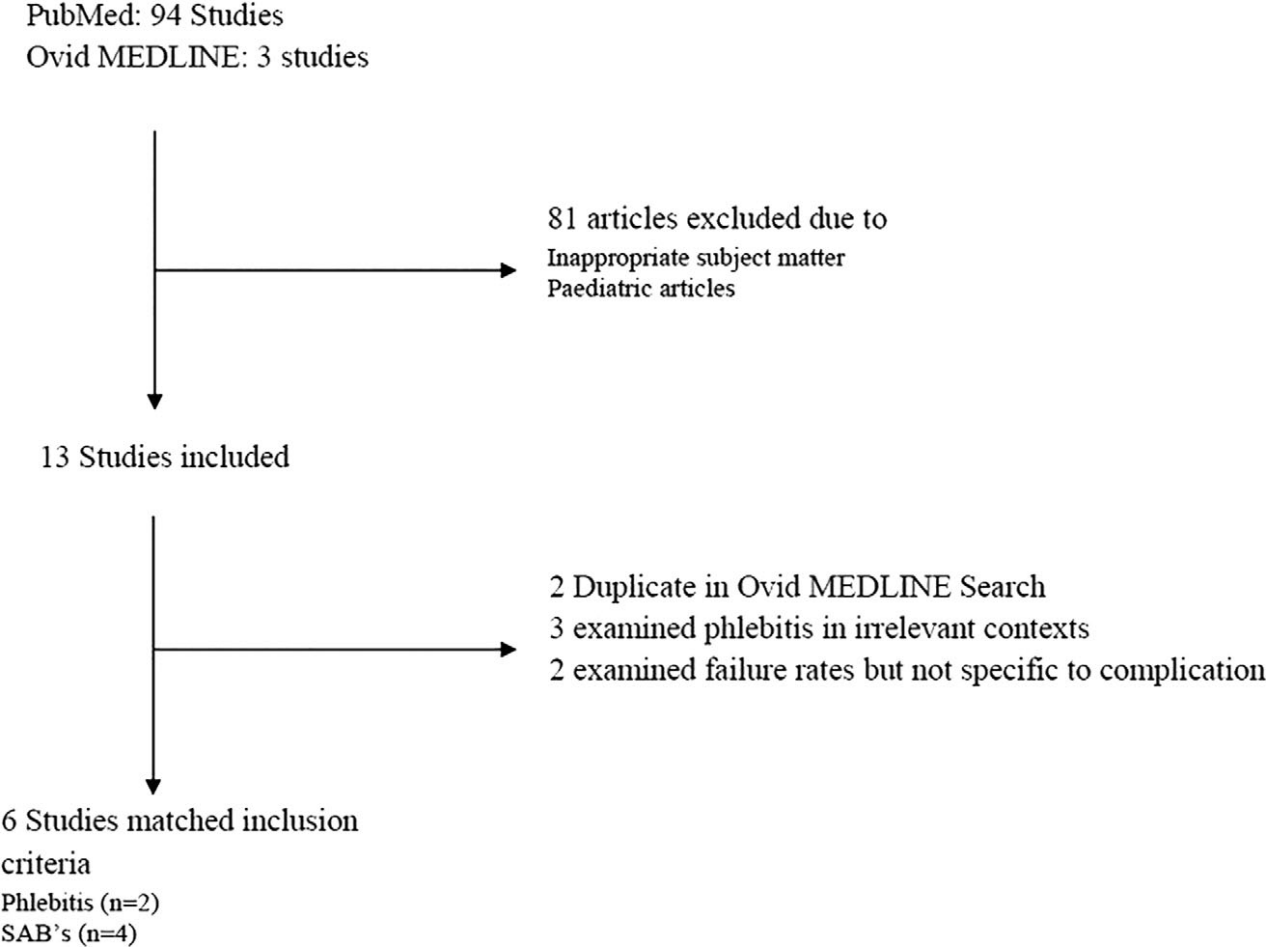
A PubMed and Ovid MEDLINE literature search workflow describing exclusion and retention criteria for studies on cannula complication data. Search terms included in Appendix S1.

To assess the prevalence of complications caused by cannula insertions, inclusion criteria included adult patients, cannula inserted in ED and bacteraemia attributed to cannula use. Secondary inclusion criteria included hospital LoS and other complications (Fig. 2). Complications that increased LoS included phlebitis and bacteraemia. The literature search revealed that phlebitis occurred in 17–31% of cannulations.^12^, ^14^, ^15^ According to Campbell,^15^ phlebitis can extend hospital stay anywhere from 2 to 5 days, however, as a conservative estimate, we chose to halve the admission extension calculated by Campbell^15^ for this complication, estimating an extra 1 day in hospital. This is to account for missed therapeutics (e.g. antibiotics) as a result of cannula complications.^16^ As such, an episode of phlebitis could increase hospital cost by A$2231 per person.^7^ We factored admission rates into the calculation because phlebitis is usually diagnosed in admitted patients, and approximately 31% of patients from ED were admitted.^9^ Bacteraemia events were found to increase LoS by 3 weeks (~15 working days) at a cost A$33 366 per episode.^5^, ^10^ The AIHW reported 1428 episodes of SAB occurred in 2019/2020, but did not state how many of these are attributable to PIVCs.^9^ A previous study found in the review reported that 23.5% of hospital‐acquired SAB cases were attributed to cannula.^5^

**Figure 3:**
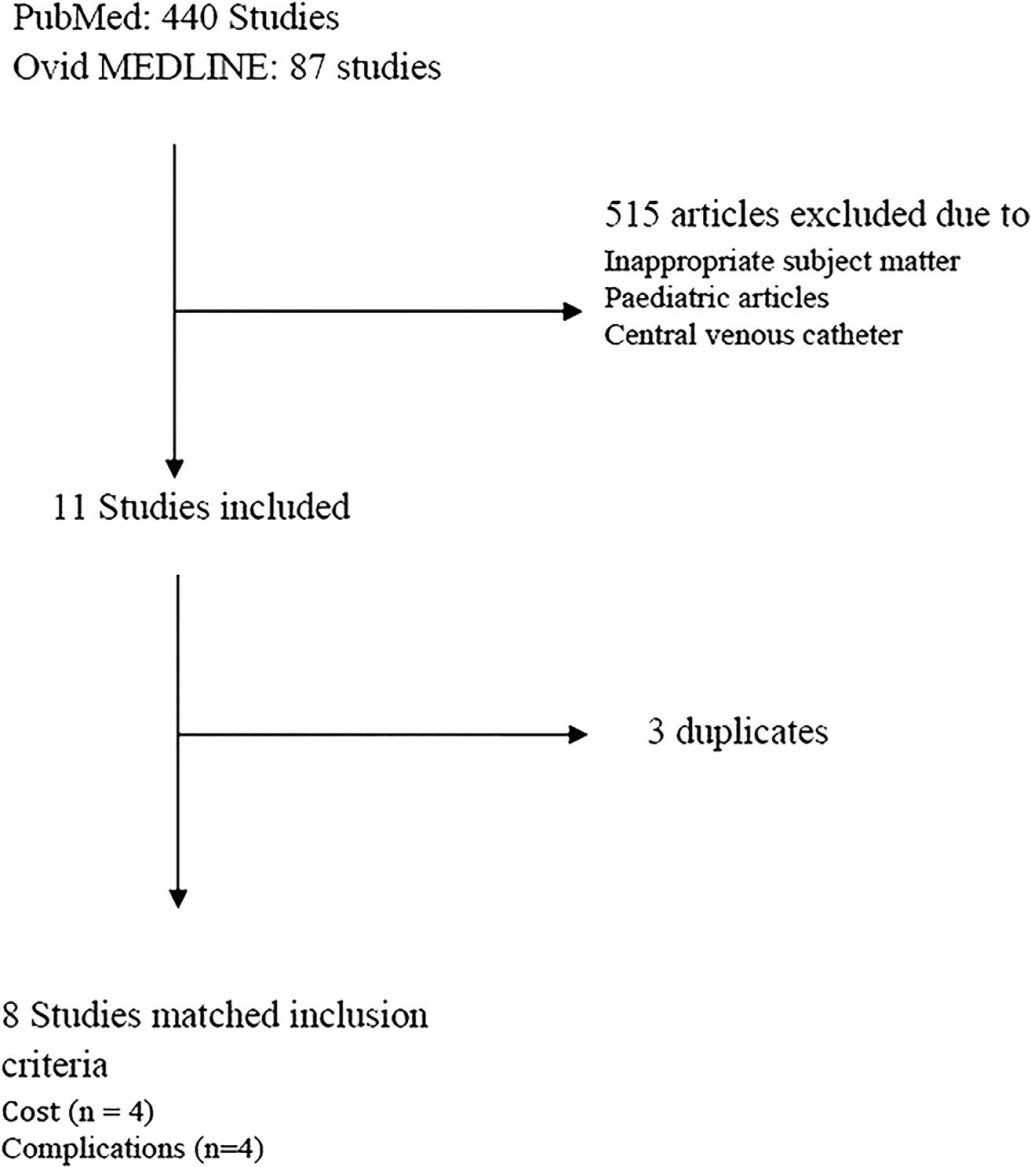
A PubMed and Ovid MEDLINE literature search workflow describing exclusion and retention criteria for studies on cannula cost and non‐infective complications.

Patient‐centred cost integrated complications that typically do not increase LoS, however, cause significant patient distress (Fig. 3). Examples of these include occlusion and infiltration, and dislodgement which, according to the literature search, occurred at rates of 14% and 10%, respectively.^15^ Management of these complications typically requires cannula replacement, so the estimated number of cannulas with these complications was calculated and multiplied by the cost of a new cannula to estimate the cost of replacing them. Loss of productivity was included in ‘patient‐centred cost’, in the form of business days absent from work as ‘sick days’ added to the patients' hospital stay because of complications such as phlebitis and SAB. As such, the number of ED attendances was narrowed to include those below the average retirement age in Australia, which is approximately 63 years old.^17^ Each ‘sick day’ was estimated to cost A$355 per day.^18^

## Results

National data sets recorded 6.6 million attendances to public EDs across Australia in the financial year 1 July 2019 to 30 June 2020.^9^ With the rate of cannula insertion found to be 36.0–42.1%, this equates to 2.3–2.7 million cannulas being inserted in this period. Of those admitted, 17–31% developed phlebitis which corresponds to 121 000–259 000 cannulas. Of the 1428 episodes of SAB recorded from 1 July 2019 to 30 June 2020, approximately 23.5% were because of cannulas, resulting in 335 SAB infections. From the number of inserted cannulas, 14% developed infiltration or occlusion and 10% became dislodged resulting in 322 000–378 000 infiltrated/occluded cannula and 230 000–270 000 dislodgements.

Equipment cost of cannulation was estimated to total A$17.23 million (Table 1), with approximately A$7.42 million of this attributed to PIVCs which remain unused (Table 2). Staff cost of PIVCs inserted was estimated to cost A$42.93 million per year (Table 1), with approximately A$18.48 million of this total related to unused PIVCs (Table 2).

**TABLE 1:** Estimated national cost of peripheral intravenous cannula use in Australia, including ‘sick day’ cost

Category	Lower range (A$ mil)	Upper range (A$ mil)	Average (A$ mil)
Equipment cost	15.89	18.58	17.23
Staff cost	39.58	46.29	42.93
Phlebitis	279.36	595.73	437.52
SAB cost	11.17	N/A	11.17
Occlusion/infiltration and dislodgment	12.89	15.13	14.01
Sick day cost – phlebitis	44.45	94.79	69.62
Sick day cost – SAB	1.78	N/A	1.78
Total cost to hospital	358.89	675.73	522.86
Total cost to community	43.23	94.79	71.4
Total economic impact			594.26

A$, Australian dollars; PIVC, peripheral intravenous cannula; SAB, *Staphylococcus aureus* bacteraemia.

**TABLE 2:** Estimated national cost of unused peripheral intravenous cannula use in Australia

Category	Lower range (A$ mil)	Upper range (A$ mil)	Average (A$ mil)
Unused equipment cost	5.18	9.66	7.42
Unused staff cost	12.90	24.07	18.48
Cost of unused PIVC phlebitis	91.07	309.78	200.43
Cost of unused PIVC SAB	3.64	N/A	3.64
Unused occlusion/infiltration and dislodgment	4.20	7.86	6.03
Unused PIVC phlebitis sick day	14.49	49.29	31.89
Unused SAB sick day	0.58	N/A	0.58
Total cost of unused PIVC to hospital	116.81	351.37	233.68
Total cost of unused PIVC to community	15.07	57.15	72.22
Total economic impact of unused PIVC			305.9

A$, Australian dollars; PIVC, peripheral intravenous cannula; SAB, *Staphylococcus aureus* bacteraemia.

Phlebitis has been estimated to cost A$437.52 million per year and the annual cost of PIVC‐associated SAB is A$11.17 million (Table 1). Therefore, the total cost of LoS‐prolonging complications is approximately A$484.69 million. Unused PIVCs contribute up to A$204.47 million annually to this cost (Table 2).

The cumulative figure of patient‐centred cost is A$85.41 million, this category includes ‘sick day’ cost to the community and the economic cost of reinsertion because of complications such as occlusion and infiltration, and dislodgement. ‘Sick day’ cost to patients and the community was A$71.4 million per annum. Of this, SAB sick days contribute A$1.78 million, and phlebitis ‘sick days’ cost A$69.62 million (Table 1). Unused PIVCs account for an estimated A$36.11 million of the total ‘sick day’ cost from this procedure (Table 2). The seemingly minor complications of infiltration and occlusion, and dislodgement, add approximately A$14.01 million to patient care annually.

Emergency clinicians spend approximately 27 383 days inserting PIVCs (Table 3, with 11 790 days spent inserting PIVCs that remain idle (Table 3). This time cannot be ‘saved’ or ‘reimbursed’ but represents a significant opportunity cost as it is time that could be reallocated to beneficial patient care.

**TABLE 3:** Estimated days used to insert total number of PIVCs including idle/unnecessary PIVCs

Category	Lower range (days)	Upper range (days)	Average (days)
Time cost of total PIVCs	25 245	29 522	27 383
Time cost of unused PIVCs	8229	15 351	11 790

PIVC, peripheral intravenous cannula.

In total, intravenous cannulation in the ED was estimated to cost the Australian Government A$594.26 million annually, with unused PIVCs contributing A$305.9 million to this total (Tables 1 and 2).

## Discussion

Cannula insertion in adult patients in Australian EDs costs more than A$500 million annually, with almost half of this cost attributed to unused cannula and, therefore, potentially avoidable. This represents up to 0.65% of Australia's A$80 billion healthcare expenditure in the hospital setting, according to the 2018–2019 Australian Bureau of Statistics figures.^19^ Importantly, there is an alternative, which is to avoid unnecessary cannulation. Reducing cannula placement and instead using venepuncture if required, would reduce the expenditure of cannula significantly, decreasing the risk of complications and improving patient outcomes as venepunctures have few adverse events reported.^20^

Using days lost from work as a measure for loss of productivity is consistent with other studies. A Canadian study examining the cost of peripherally inserted central venous catheters included a loss of productivity as a loss of a day's work for each extra day a patient spent in hospital.^21^

Previous research suggests that cannulation equipment alone costs A$6.4 million in Queensland, Australia, a state that represents both 20% of the total population and national ED presentations.^9^, ^22^ This indicates that our estimate of national equipment costs of A$17.23 million may be conservative.^8^, ^23^

These cost analyses have the potential to change practice and bring awareness to the complications associated with PIVC insertion. In a timely example of policy change that has recently been implemented to reduce inappropriate cannula use and complications, the Australian Commission on Safety and Quality in Health Care (ACSQHC) has implemented new clinical care guidelines for cannula management.^24^ Clinicians aim to deliver the best care to patients, but simply drawing attention to low‐value care through guidelines has been shown to be ineffective at changing clinical practice.^25^ Highlighting the safety aspects of a procedure is a more effective method to change clinical practice, hence the focus of the new ACSQHC guideline.^24^, ^25^

Patient‐centred cost has been incorporated into the analysis by examining the loss of productivity in the community. This is an important figure for patients and the community, as the cost may be significantly higher for those without stable work or leave entitlements. From a patient's perspective, having a painful cannula that is unlikely to be used, adding the additional risk of healthcare‐related complications is nonsensical. Research suggests this is a daily occurrence and occurs without shared clinician‐consumer decision‐making, largely because of cannula complacency.^6^, ^11^ Involving patients in shared decision making, allowing for open discussion on the indication, or lack thereof, for a cannula could make decreasing insertion rates possible. It has been shown that decreasing cannula insertion rates by 10–18% has been demonstrated to be both sustainable and safe.^6^, ^11^, ^26^

Future evaluation is needed to determine whether cannula insertion increases the use of intravenous medications in patients where oral formulations would have been suitable. Increased intravenous medication use would increase economic and patient costs, with such treatments associated with risks and adverse events. Future examination is also needed to determine whether the presence of a PIVC increases the frequency of blood draws, leading to iatrogenic anaemia.^27^, ^28^

There are limitations of this analysis. National data sets found on the MyHospitals website were able to provide figures on the number of adult ED attendances Australia‐wide and the cost of bed days but failed to provide other data for the calculations. To bridge these gaps, literature searches were used to develop ranges which improved the accuracy of the figures. Access to comprehensive national data sets would allow researchers to conduct far more accurate cost estimates, especially for factors which heavily influence the final figure, such as infective phlebitis. The cost of cannula insertion was determined in 2018, and whereas we were able to adjust for inflation for both the staff and equipment cost, other factors may have changed because of this time including packs available, and equipment used.^6^ The unused cannula calculations relied on unused cannula having the same infection prevalence as used cannula which may not be the case. Only cannula insertion rates and rates of use in the ED were calculated, and therefore, the figures may be limited in generalisability to the entire hospital system. Finally, the ‘sick day’ figure used data from all ages over 15, which includes patients who potentially do not work. To rectify this, the total number of ED attendances was limited to 15–64; however, the number of people on government support or other kinds of income support was not estimated.

## Conclusion

Cannula insertion represents a large economic and health cost to Australia's health system. Drawing attention to the impact of cannulas presents an opportunity to better utilise staff time to provide the most efficacious care. Healthcare in Australia is disadvantaged by the lack of national data for PIVC use and there is a failure to centrally document complications associated with these ubiquitous, but not harmless devices. There is an urgent need for improved data to drive clinical change, prevent harm and minimise cost associated with cannula use.

## Supporting information


**Appendix S1.** Search terms for individual database searches.

## Data Availability

The data that supports the findings of this study are provided in the supplementary material of this article.
