# Supplementary material for: From little things, big things grow: An exploratory analysis of the national cost of peripheral intravenous catheter insertion in Australian adult emergency care
Source: Emerg Med Australas. 2022 May 14;34(6):877–83. doi: 10.1111/1742-6723.14009 (PMC9790706; doi:10.1111/1742-6723.14009)
Supplement: Supplementary file 1 — Appendix S1. Search terms for individual database searches. [file EMM-34-877-s001.docx]

**Appendix S1.** Search terms for individual database searches

| **PIVC Search** | *peripheral intravenous catheter* or *peripheral venous catheter* or *PVC* or *PIVC* or *cannula** or *drip**  AND *rates of insertion* or *insertion rate* or *rates of use* or *number inserted* or *number of insertions* or *number insert** | *peripheral intravenous catheter* or *peripheral venous catheter** or *PVC* or *PIVC* or *cannula** or *drip**  AND *rate* of insertion* or *insertion rate* or *rate of use* or *number inserted* or *number of insertion** or *number insert** or *amount inserted*  NOT *p*ediatric* or *high flow*  AND *emergency department* or *emergency room* or *ED* or *acute care* |
| --- | --- | --- |
| **SAB Search** | *peripheral intravenous catheter* or *peripheral venous catheter* or *PVC* or *PIVC* or *cannula* or *drip*  AND *staphylococcus aureus bacter*emia* or *SAB* or *S aureus infection* or *blood infection** or *infection in blood* or *bacteria in blood* | *peripheral intravenous catheter* or *peripheral venous catheter* or *PVC* or *PIVC* or *cannula** or *drip**  AND *staphylococcus aureus bacter*emia* or *SAB* or *S aureus infection* or *blood infection* or *infection in blood* or *bacteria in blood*  AND *emergency department* or *emergency room* or *ED* or *acute care*  NOT *p*ediatric* or *high flow* or *nasal* or *central line* |
| **Cost Search** | *peripheral intravenous catheter* or *peripheral venous catheter* or *PVC* or *PIVC* or *cannula** or *drip* or *venous access*  AND *cost* or *price* or *cost per unit* or *price per unit* or *economic analys*s* or *cost analys*s* or *economic evaluation* or *cost evaluation* or *staff cost* | *peripheral intravenous catheter** or *peripheral venous catheter* or *PVC* or *PIVC* or *cannula** or *drip* or **vascular access*  AND *cost* or *cost of consumable** or *staff cost* or *staff time* or *cost of staff time* or *expenditure* or *hospital price**  AND *failure* or *cost of failure* or *complication**  AND *rate* of insertion* or *insertion rate** or *rate* of use* or *number inserted* or *number* of insert** or *number insert** or *amount inserted*  NOT *high flow* or *nasal* or *high flow nasal cannula* |
